# Basal interferon signaling and therapeutic use of interferons in controlling rotavirus infection in human intestinal cells and organoids

**DOI:** 10.1038/s41598-018-26784-9

**Published:** 2018-05-29

**Authors:** Mohamad S. Hakim, Sunrui Chen, Shihao Ding, Yuebang Yin, Aqsa Ikram, Xiao-xia Ma, Wenshi Wang, Maikel P. Peppelenbosch, Qiuwei Pan

**Affiliations:** 1 Department of Gastroenterology and Hepatology, Erasmus MC-University Medical Center and Postgraduate School Molecular Medicine, Rotterdam, The Netherlands; 2 Department of Microbiology, Faculty of Medicine, Universitas Gadjah Mada, Yogyakarta, Indonesia; 3 Atta-Ur-Rahman School of Applied Biosciences, National University of Science and Technology, Islamabad, Pakistan; 4 Key Laboratory of Bioengineering and Biotechnology of State Ethnic Affairs Commission; Engineering and Technology Research Center for Animal Cell, College of Life Science and Engineering, Northwest University for Nationalities, Lanzhou, China

## Abstract

Rotavirus (RV) primarily infects enterocytes and results in severe diarrhea, particularly in children. It is known that the host immune responses determine the outcome of viral infections. Following infections, interferons (IFNs) are produced as the first and the main anti-viral cytokines to combat the virus. Here we showed that RV predominantly induced type III IFNs (IFN-λ1), and to a less extent, type I IFNs (IFN-α and IFN-β) in human intestinal cells. However, it did not produce detectable IFN proteins and thus, was not sufficient to inhibit RV replication. In contrast, we revealed the essential roles of the basal IFN signaling in limiting RV replication by silencing *STAT1*, *STAT2* and *IRF9* genes. In addition, exogenous IFN treatment demonstrated that RV replication was able to be inhibited by all types of IFNs, both in human intestinal Caco2 cell line and in primary intestinal organoids. In these models, IFNs significantly upregulated a panel of well-known anti-viral IFN-stimulated genes (ISGs). Importantly, inhibition of the JAK-STAT cascade abrogated ISG induction and the anti-RV effects of IFNs. Thus, our study shall contribute to better understanding of the complex RV-host interactions and provide rationale for therapeutic development of IFN-based treatment against RV infection.

## Introduction

Rotavirus (RV) is a member of the *Reoviridae* family that primarily infects mature enterocytes of the small intestinal villi. However, it can spread systematically to cause viremia and infect multiple organs^1^. RV is the most frequent agent of severe dehydrating diarrhea episodes in children under five years of age^2^. Before introduction of RV vaccines, RV caused 9.8 billion of severe diarrhea episodes and 1.9 billion diarrhea-related deaths worldwide, with the highest burden in southeast Asian and African countries^3^. The incidence is lower especially in countries that have introduced oral RV vaccination^4^.

Innate immune responses are the first line defenses critical to battle RV infection^5^. Recognition of RV viral proteins and double-stranded RNA by the host induces the production of cytokines, including interferons (IFNs)^6^. IFNs are potent anti-viral cytokines classified into three different groups, type I (IFN-α, IFN-β, IFN-δ, and others), type II (IFN-γ) and type III (IFN-λ1, IFN-λ2 and IFN-λ3) IFNs^7,8^. Some members are widely used in the clinic for treating viral infections or malignancy; whereas others are at stages of clinical development. Even though they bind to distinct receptors, they signal through a common, classical Janus kinase signal transducer and activator of transcription (JAK-STAT) pathway^8,9^.

Once activated, STAT1 and STAT2 are phosphorylated and bind IFN regulatory factor 9 (IRF9) to form IFN stimulated gene factor 3 complex (ISGF3). ISGF3 subsequently translocates to the nuclues, leading to induced transcription of hundreds IFN-stimulated genes (ISGs) which cooperatively establish an anti-viral state against various types of viruses^10^. Furthermore, IFN induction following RV recognition is essential to promote the development of adaptive, B-cell mediated immune responses^11^. On the other hand, however, RV has developed effective strategies to evade the host immune response^12^. RV can inhibit IFN production in the infected cells^13^ and also block the action of STAT1 and STAT2 proteins^14^. Viral nonstructural protein NSP1-mediated IFN inhibition has been shown to be associated with different levels of RV replication in primary mouse cells^15^.

Detectable levels of IFN-α^16^ and IFN-γ^17,18^ were documented in chidlren with acute RV diarrhea, suggesting their roles in the disease pathogenesis. Indeed, early *in vitro*^19–21^ and animal studies in calves^22^ and piglets^23^ demonstrated anti-RV effects of both type I and II IFNs. However, in the murine models of homologous RV infection, administration of type I (IFN-α and IFN-β) and II (IFN-γ) IFNs failed to protect the mice against RV infection^24^. In addition, mice deficient in type I or II IFN receptor signaling controlled RV infection as wild-type mice^24,25^. These results suggest a minor role of type I and II IFNs in controlling RV infection in mice. Interestingly, a more prominent role of type III IFNs was demosntrated in the mouse model^25,26^. Administration of IFN-λ conferred better protection against RV infection than IFN-α/β^25^.

Because animal models do not always recapitulate the responsiveness in human, we therefore comprehensively assessed the role of endogenous and the therapeutic IFNs on RV infection in human intestinal cell line and primary intestinal organoids. We found that the basal JAK-STAT cascade is effective in restraining RV infection. Furthermore, RV is sensitive to inhibition by all three types of IFNs in both models. Our results strengthen the evidence of essential roles of IFN pathway in protecting the host against viral infection.

## Results

### RV infection modulates *IFN* gene expression

First, we investigated whether RV SA11 modulates the expression of the three types of *IFN* genes. Human intestinal Caco2 cells were infected with RV SA11 for 48 hours. An effective replication was shown by an increase in intracellular RNA level as well as secreted rotavirus in culture medium (Supplementary Fig. S1). In addition, immunofluorescence staining showed VP6-positive Caco2 cells at 48 hours after infection, indicating productive replications (Supplementary Fig. S2).

**Figure 1:**
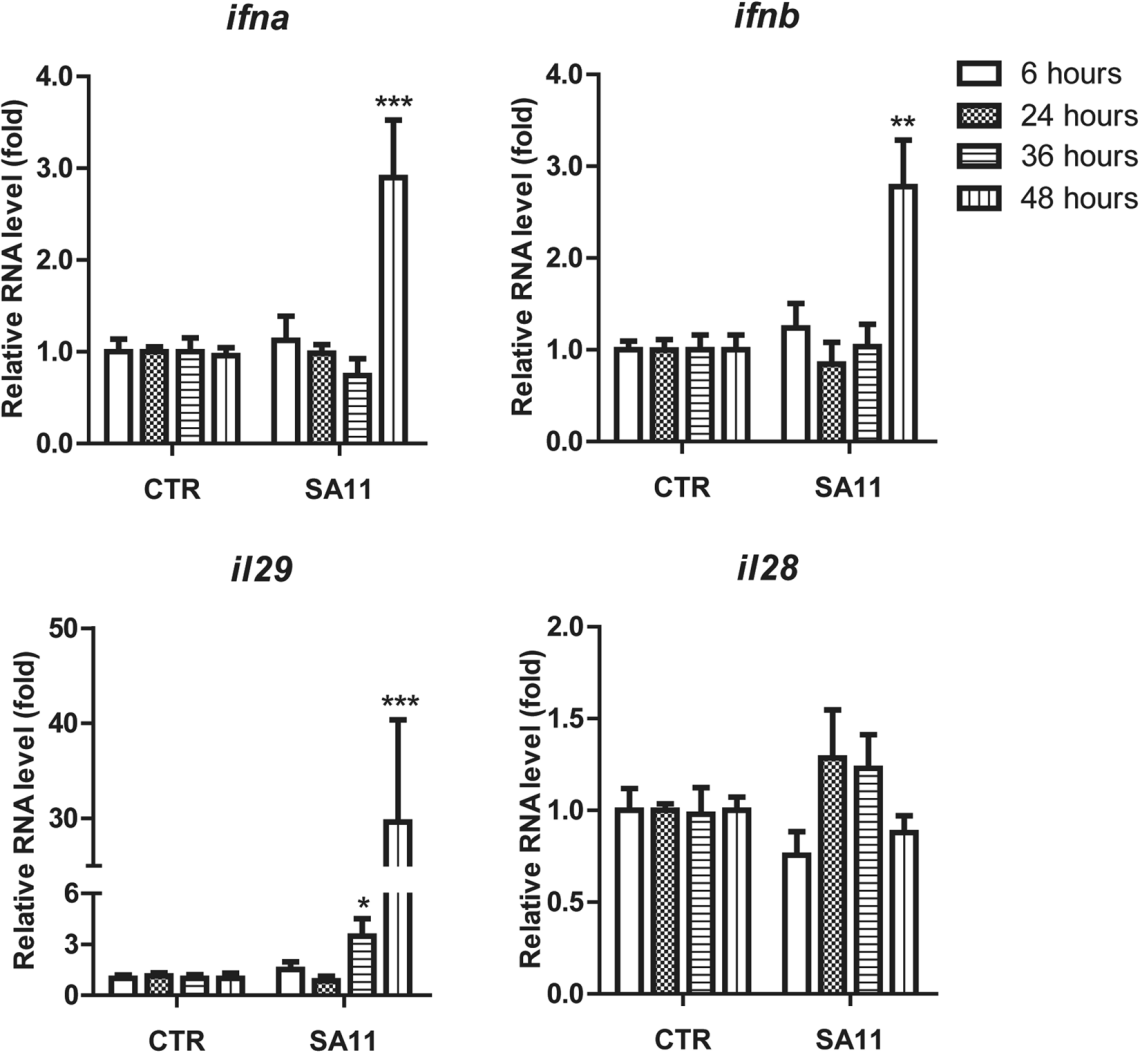
RV infection modulates IFN gene expression in Caco2 cells. Caco2 cells were infected with RV SA11. Relative RNA levels of *ifna*, *ifnb*, *il29* (IFN-λ1) and *il28* (IFN-λ2 and IFN-λ3) genes were examined at 6, 24, 36 and 48 hours post infection as compared to uninfected cells. Data were normalized to GAPDH and presented as means ± SEM. (n = 3 independent experiments with each of 3–4 replicates; **P < 0.01; ***P < 0.0001).

Relative RNA levels of *ifna*, *ifnb*, *ifng*, *il29* (IFN-λ1) and *il28* (IFN-λ2/IFN-λ3) genes were examined and compared to uninfected cells at 6, 24, 36 and 48 hours post infection. As shown in Fig. 1, RV infection had no major effect on the gene expression at 6 and 24 hours post-infection. At 36 hours after infection, only *il29* gene expression was notably increased by 3.4 ± 1.0 (*P* < 0.05) fold. Importantly, at 48 hours after infection, the expression of *ifna* and *ifnb* genes were significantly increased by 2.8 ± 0.6 (*P* < 0.001) and 2.8 ± 0.5 (P < 0.01) fold, respectively. A profound upregulation was observed on *il29* by 29.6 ± 10.7 fold (*P* < 0.001). No difference was found on *il28* gene expression. The expression level of *ifng* gene was undetectable (data not shown). Together, our findings showed that RV SA11 infection preferentially induced *il29* (IFN-λ1) gene expression in Caco2 cells.

### The increased expression of IFN genes does not result in production of detectable IFN protein and was not sufficient to limit RV SA11 replication

**Figure 2:**
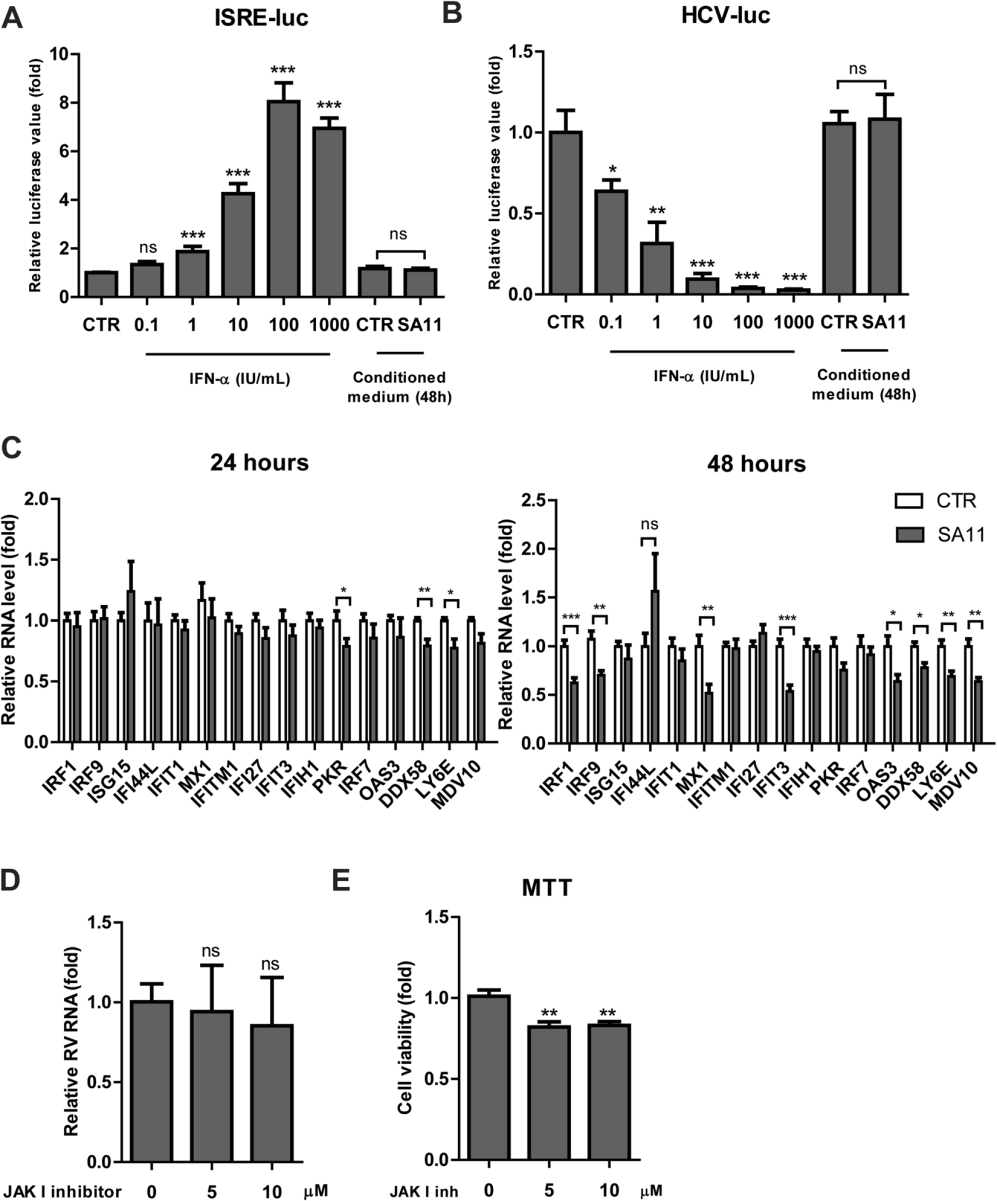
The increased expression of IFN genes does not result in production of detectable IFN protein and was not sufficient to limit RV SA11 replication. IFN production bioassay was performed in ISRE-luciferase (**A**) and HCV-luciferase (**B**) cell lines which are highly sensitive to IFN treatment. Conditioned medium derived from 48 hours post-RV infection on Caco2 cells was used (n = 3 independent experiments with each 2–3 replicates). (**C**) Caco2 cells were infected with RV SA11. Relative RNA levels of IFN-stimulated genes (ISGs) were examined at 24 and 48 hours post-infection as compared to uninfected cells (n = 3 independent experiments with each 2–3 replicates). (**D**) *Pan*-JAK I inhibitor had no effects on RV replication (n = 3 independent experiments with each 2–3 replicates). (**E**) *Pan*-JAK I inhibitor did not affect cell viability as determined by MTT assay (OD_490_ value) at 48 hours of treatment (n = 3 independent experiments with each 2 replicates). Data were presented as means ± SEM., **P < 0.01; ***P < 0.001; ns, not significant.

To examine whether the increased expression of IFN genes result in IFN production in our cell culture system, we collected the conditioned medium (supernatant) derived from control and SA11-infected Caco2 cells at 48 hours post-infection. Then, we performed IFN production bioassay by adding the conditioned medium into two highly IFN sensitive cell lines, Huh7-based ISRE-luciferase and HCV-luciferase reporter cell lines. As shown in Fig. 2A, the supernatant from SA11-infected Caco2 cells was not capable of stimulating ISRE reporter, while as low as 1 U/mL of IFN-α significantly induced ISRE-luc by 1.8 ± 0.2 fold (*P* < 0.001). Consistently, it was not able to diminish HCV replication, although as low as 0.1 IU/mL of IFN-α considerably reduced HCV-luc by 40 ± 7% fold (*P* < 0.001) (Fig. 2B).

The induced expression of IFN-α and IFN-λ1 proteins can signal via autocrine and paracrine manner to stimulate ISGs expression. Therefore, to confirm our previous findings from ISRE-luciferase cell lines, we also examined ISG expression in SA11-infected Caco2 cells. Despite a clear induction of IFN genes, we observed no upregulation of ISGs, both at 24 and 48 hours post-infection. At 48 hours, some ISGs were significantly downregulated by SA11 infection, including IRF1, IRF9, MX1 and IFIT3 (Fig. 2C).

To rule out the possibility that endogenous IFN produced (if any) following RV infection is sufficient to restrict RV replication, we investigated whether the inhibition of JAK proteins, the downstream elements of IFN receptor, influences RV replication. Treatment of JAK I inhibitor at 5 and 10 μM had no effect on RV replication (Fig. 2D). At those concentrations, the drug did not influence the cell viability as determined by MTT assay (Fig. 2E). Collectively, our results demonstrated that the increased expression of IFN genes during RV infection did not result in IFN production and consequently, was not sufficient to limit RV replication.

### The basal IFN signaling is necessary to restrict RV replication

Although no functional level of IFN is induced by RV infection, we delineated the role of basal IFN signaling in regulating RV replication. ISGF3 that consists of STAT1, STAT2 and IRF9 is a central complex dictating the IFN signaling. We have previously reported that in the absence of IFN stimulation, unphosphorylated ISGF3 drives constitutive ISG expression in homeostatic condition and is critical to provide immunity against hepatitis C (HCV) and E (HEV) virus infections^27^.

**Figure 3:**
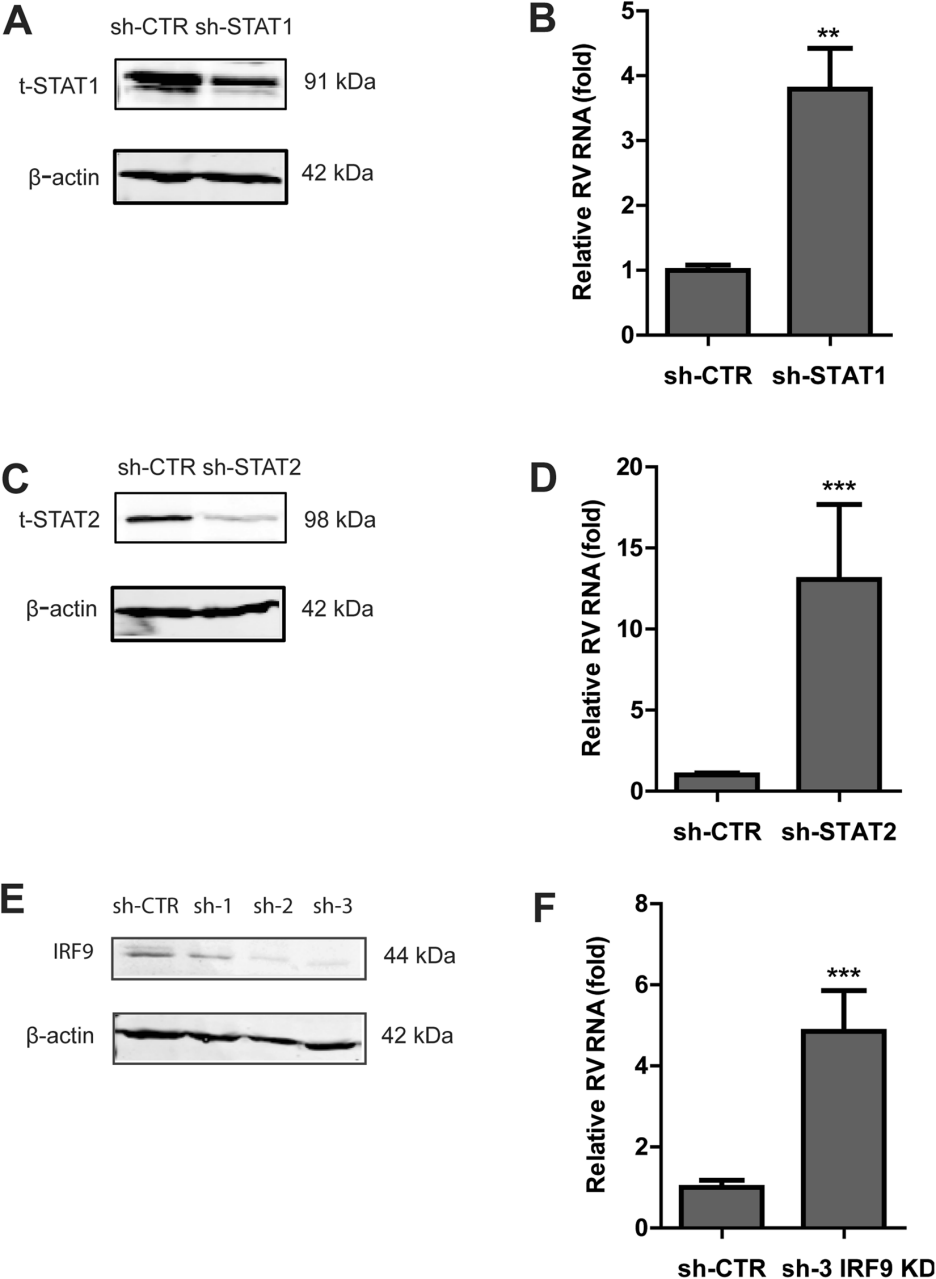
The key component of ISGF3 complex is necessary to restrict RV replication. (**A**) STAT1 knockdown by lentiviral shRNA vectors. Western blot analysis confirms a successful knockdown of total STAT1 protein. (**B**) Correspondingly, knockdown of STAT1 led to a significant increase of RV replication (n = 3 independent experiments with 3–4 replicates each). (**C**) Knockdwon of STAT2 by lentiviral shRNA vectors. Western blot analysis shows a potent decrease of total STAT2 protein level. (**D**) Similarly, silencing of STAT2 resulted in a prominent increase of RV RNA level (n = 3 independent experiments with 3–4 replicates each). (**E**) IRF9 knockdwon by lentiviral shRNA vectors. Western blot analysis shows a potent decrease of IRF9 protein level. (**F**) Knockdown of IRF9 led to a notable increase of RV replication (n = 3 independent experiments with 3–4 replicates each). Data were presented as means ± SEM., **P < 0.01; ***P < 0.001.

First, we transduced Caco2 cells with a lentiviral vector expressing STAT1- and STAT2-specific shRNA. A successful knockdown was shown in Fig. 3A,C. Supplementary Fig. S3 showed the quantification of knockdown efficiency. Importantly, shRNA-mediated STAT1 and STAT2 knockdown resulted in an increased RV replication by 3.8 ± 0.6 fold (*P* < 0.01) (Fig. 3B) and 13 ± 4.6 (*P* < 0.001) (Fig. 3D), respectively.

Next we investigated the role of IRF9. Two of three tested IRF9-specific shRNA (sh-2 and sh-3) exert a potent gene silencing capacity (Fig. 3E and Supplementary Fig. S3). Consistently, IRF9 KD led to 4.8 ± 1.0 fold elevation of RV replication as compared to sh-CTR transfected cells (*P* < 0.001) (Fig. 3F). Altogether, these results indicate that the integrity of ISGF3 complex is required to provide basal immunity against RV infections.

### RV SA11 is sensitive to IFN treatment in human intestinal Caco2 cells and primary intestinal organoids

**Figure 4:**
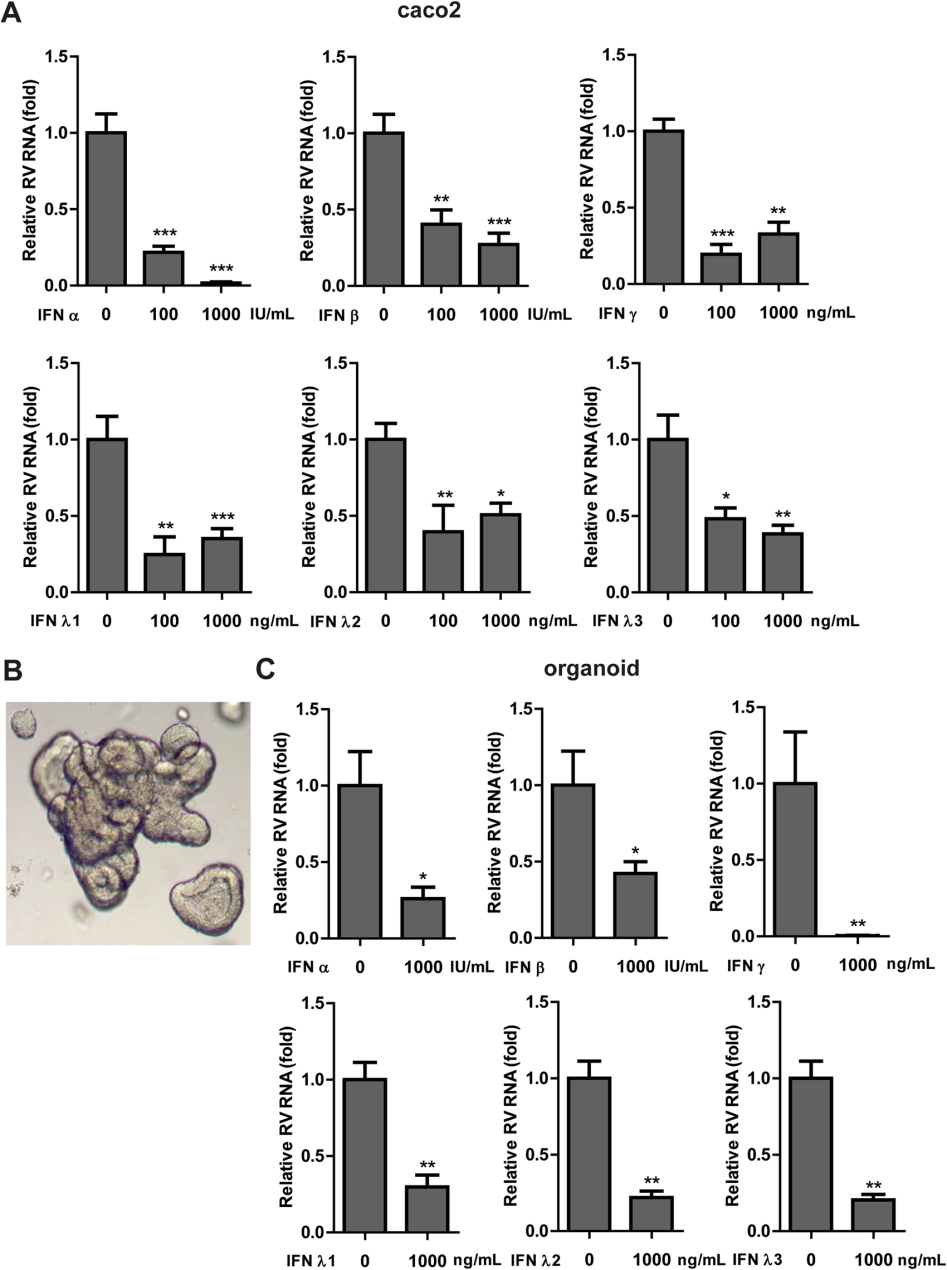
Exogenous treatment of type I, II and III IFNs inhibits RV SA11 infection. Antiviral activity of IFNα, IFNβ, IFNγ, IFNλ1, IFNλ2 and IFNλ3 treatment against RV SA11 infection on (**A**) Caco2 cells (n = 2–3 independent experiments with each of 3–4 replicates) and (**C**) organoids (n = 3 independent experiments with each of 2–3 replicates) at 48 hours after infection. Data were presented as means ± SEM., *P < 0.05; **P < 0.01; ***P < 0.001. (**B**) A representative picture of the morphology of human small intestinal organoid at day 4 post embedding in Matrigel. The organoid used in this experiment was derived from one individual (P1).

Since we did not find a significant role of endogenous IFN in restricting RV replication, we then investigated whether RV was sensitive to exogenous IFN treatment (Fig. 4A). Treatment of SA11-infected Caco2 cells with 100 and 1000 IU/mL of IFNα resulted in a potent inhibition of RV replication by 79 ± 4% (*P* < 0.001) and 98 ± 0.7% (*P* < 0.001) as measured in total RNA levels. Similarly, IFNβ treatment at 100 and 1000 IU/mL dose-dependently inhibited total viral RNA levels by 60 ± 9% (*P* < 0.01) and 73 ± 7% (*P* < 0.001). Type II IFNs also strongly reduced RV replication, although the effects were not dose-dependent. At concentration of 100 and 1000 ng/mL, IFNγ restricted RV replication by 81 ± 6% (*P* < 0.001) and 68 ± 7% (*P* < 0.01). Analysis of intra and extracellular RNA levels also demonstrated the inhibition of RV replication by type I and II IFNs in Caco2 cells (Supplementary Fig. S4).

Next, we investigated anti-RV effects of type III IFNs. Treatment of SA11-infected Caco2 cells with 100 and 1000 ng/mL of IFNλ1 resulted in a notable restriction of RV replication by 76 ± 11% (*P* < 0.01) and 65 ± 6% (*P* < 0.001). Inhibition of RV replication was also observed with IFNλ2 treatment. At concentration of 100 and 1000 ng/mL, IFNλ2 decreased RV replication by 61 ± 17% (*P* < 0.01) and 50 ± 7% (*P* < 0.05), respectively. At a similar concentration, IFNλ3 significantly dimished RV replication by 52 ± 7% (*P* < 0.05) and 62 ± 6% (*P* < 0.01). Analysis of intra and extracellular RNA levels also demonstrated the effects of type III IFNs in limiting rotavirus replication in Caco2 cells (Supplementary Fig. S4).

To further confirm the effects of various types of IFNs that we observed in the conventional 2D cell culture system, we employed a 3D culture model of primary intestinal organoids derived from one individual (P1) to more closely mimick the physiological situation *in vivo* (Fig. 4B). Treatment of SA11-infected organoid with 1000 IU/mL of IFNα and IFNβ led to a significant reduction of intracellular viral RNA levels by 73.9 ± 7.5% (*P* < 0.05) and 57.8 ± 7.7% (*P* < 0.05), respectively. Surprisingly, the inhibition by IFNγ was more pronounced. Treatment of SA11-infected organoid with 1000 ng/mL of IFNγ significantly reduced intracellular viral RNA levels by 99.5 ± 0.1% (*P* < 0.01) (Fig. 4C). Similarly, treatment with 1000 ng/mL of IFNλ1, IFNλ2 or IFNλ3 significantly diminished intracellular viral RNA levels by 70.2 ± 7.6% (*P* < 0.01), 78.2 ± 4.2% (*P* < 0.01) and 79.6 ± 3.6% (*P* < 0.01), respectively (Fig. 4C). Quantification of extracellular (secreted) RNA levels also showed a notable reduction of viral production (Supplementary Fig. S5). To confirm these findings, we obtained primary intestinal organoids from the second individual (P2). Similarly, treatment of SA11-infected organoid with IFNα (1000 IU/mL), IFNγ (1000 ng/mL) and IFNλ1 (1000 ng/mL) significantly reduced total viral RNA levels by 74.6 ± 5.1% (*P* < 0.01), 85.5 ± 4.4% (*P* < 0.01) and 71.4 ± 10.5% (*P* < 0.01), respectively (Supplementary Fig. S6A). Collectively, these results suggest that all type of IFNs effectively inhibit RV replication, both in 2D and 3D culture model system.

### Sensitivity of patient-derived RV strains to type I, II and III IFNs

**Figure 5:**
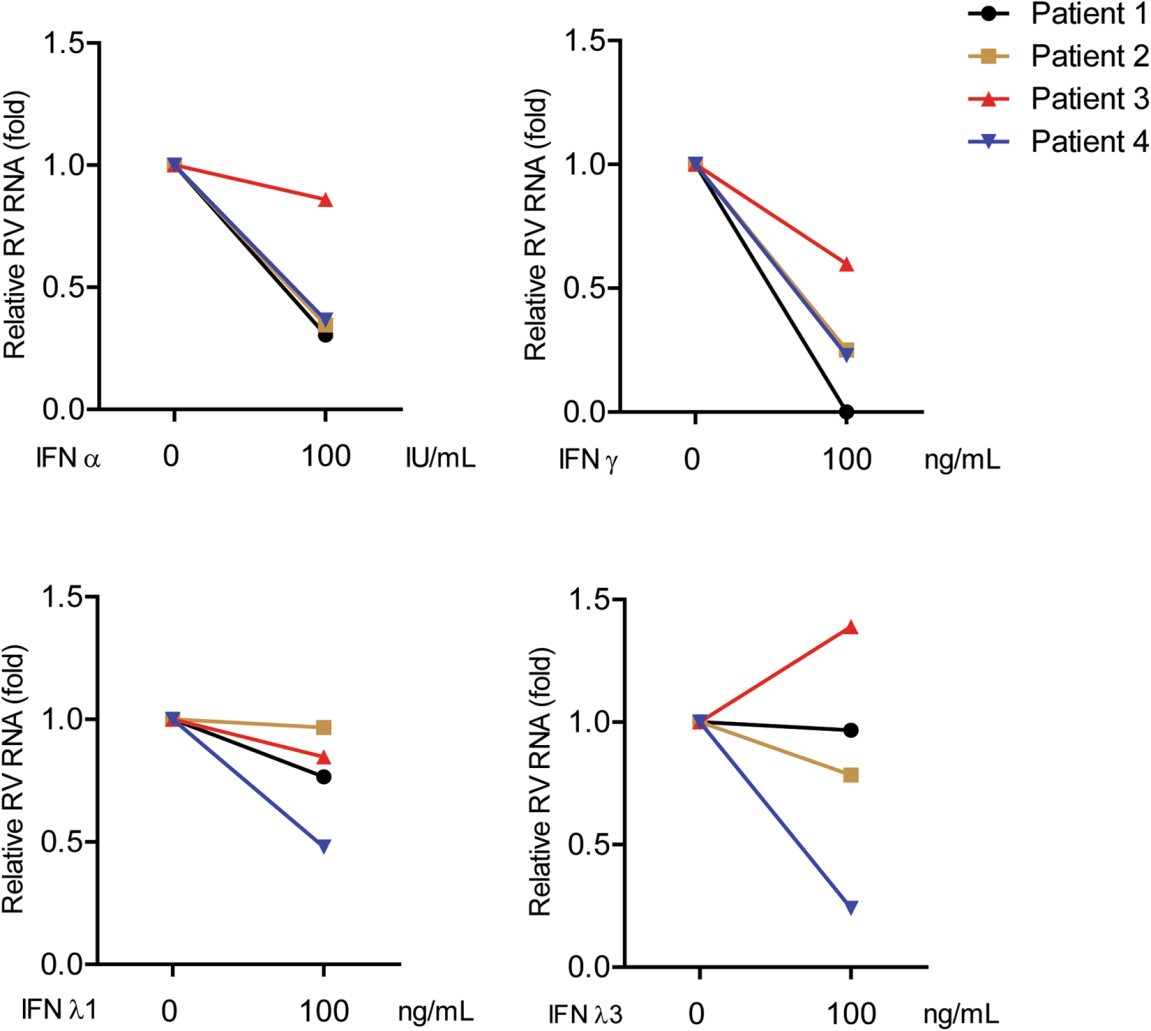
Sensitivity of patient-derived RV strains to type I, II and III IFNs. Caco2 cells were infected with four different patient-derived RV strains (G1P[8]) and treated with IFNα 100 IU/mL (as representative of type I IFN), IFNγ 100 ng/mL (type II IFN), IFNλ1 and IFNλ3 100 ng/mL (as representative of type III IFN). Distinct sensitivity was observed among these patient-derived RV samples. Human RV RNA levels were quantified by qRT-PCR at 48 hours post-infection and normalized to a reference gene GAPDH. The data are derived from an experiment for multiple patient-derived RV strains.

Next, we evaluated the sensitivity of patient-derived RV strains against different types of IFNs. We treated human RV (G1P[8]) with IFNα 100 IU/mL (as representative of type I IFN), IFNγ 100 ng/mL (type II IFN), IFNλ1 and IFNλ3 100 ng/mL (as representative of type III IFN) for 48 hours. Three out of four samples were sensitive to the inhibition by IFNα and all samples were inhibited by IFNγ (Fig. 5). Interestingly, only one sample which is sensitive to type III IFN treatment. Collectively, our data suggest that type I and II IFN more efficiently inhibit the replication of human RV strains as compared to type III IFN.

### Induction of the known antiviral ISGs by all three types of IFNs

**Figure 6:**
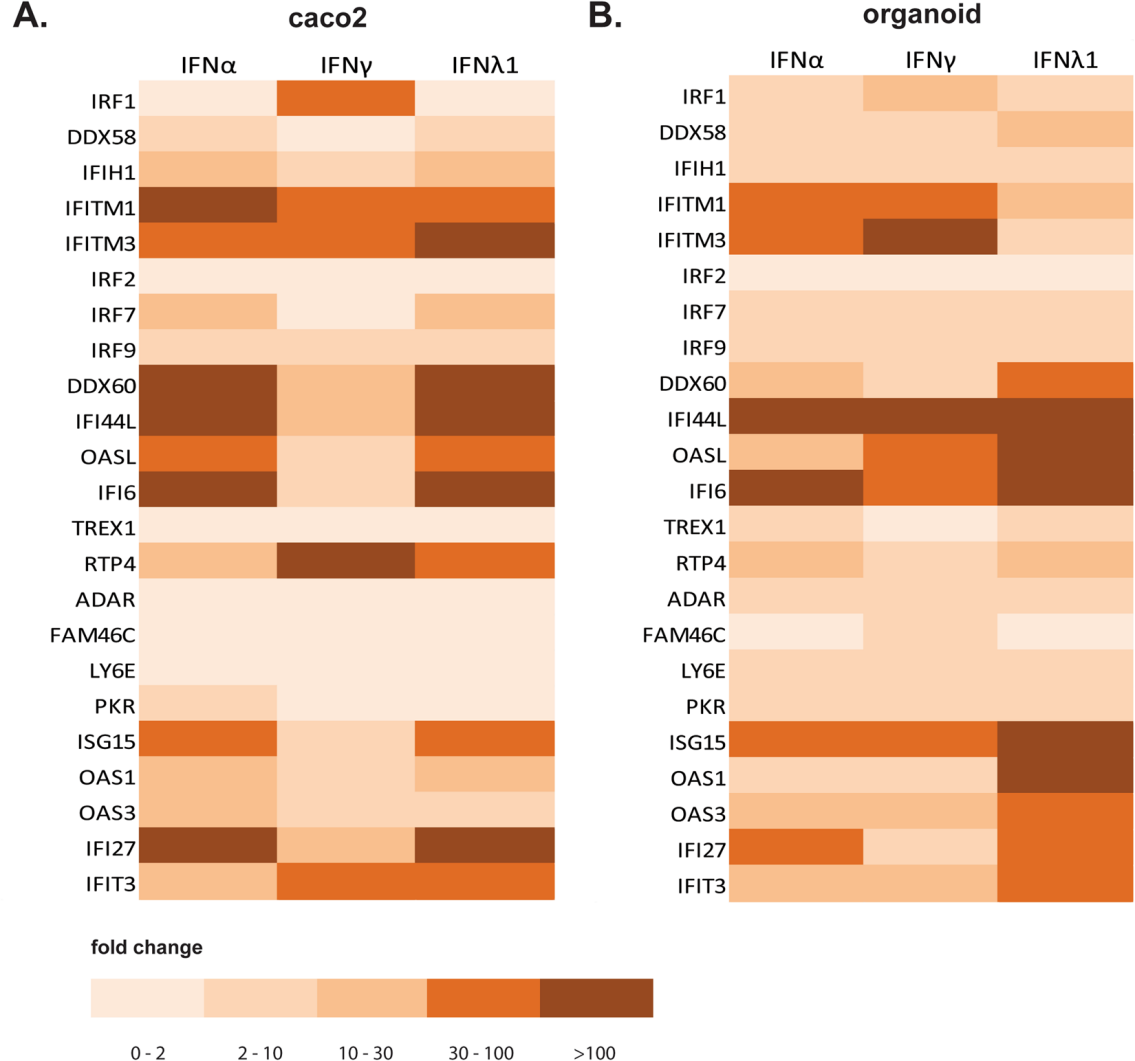
ISG induction by type I, II and III IFNs on Caco2 cells and organoids. Caco2 cells (**A**) and organoids (**B**) were stimulated with IFNα 1000 IU/mL (as representative of type I IFN), IFNγ 1000 ng/mL (type II IFN) and IFNλ1 1000 ng/mL (as representative of type III IFN) for 24 hours. The expression levels of several ISGs were measured by qRT-PCR. The organoid used in this experiment was derived from one individual (P1).

The observed anti-RV activity of type I, II and III IFNs prompted us to investigate whether all types of IFNs effectively induce the expression of known anti-viral ISGs in Caco2 cells and organoid. Although there are hundreds of ISGs, only a subset have broad or targeted antiviral effects^28^. We have selectively investigated the expression of those known antiviral ISGs. Indeed, treatment of Caco2 cells with recombinant human IFN-α (1000 IU/mL), IFN-γ (1000 ng/mL) and IFN-λ1 (1000 ng/mL) for 24 hours induced the expression a panel of ISGs (Fig. 6A). Similarly, they also efficiently induced ISGs in organoids derived from both P1 (Fig. 6B) and P2 (Supplementary Fig. S6B). Interestingly, we observed a variation of the type and extent of ISG induction with different IFN treatment. For example, IRF1 and RTP4 were more induced by IFN-γ as compared to IFN-α in Caco2 cells. In contrast, DDX60 and IFI6 were more induced by IFN-α as compared to IFN-γ (Fig. 6A). In organoid (P1), several ISGs were more efficiently induced by IFN-λ1 as compared to IFN-α and IFN-γ, including OASL, ISG15 and OAS1 (Fig. 6B).

### Inhibition of JAK-STAT signaling abrogates the anti-RV activity of IFN-α and IFN-γ

**Figure 7:**
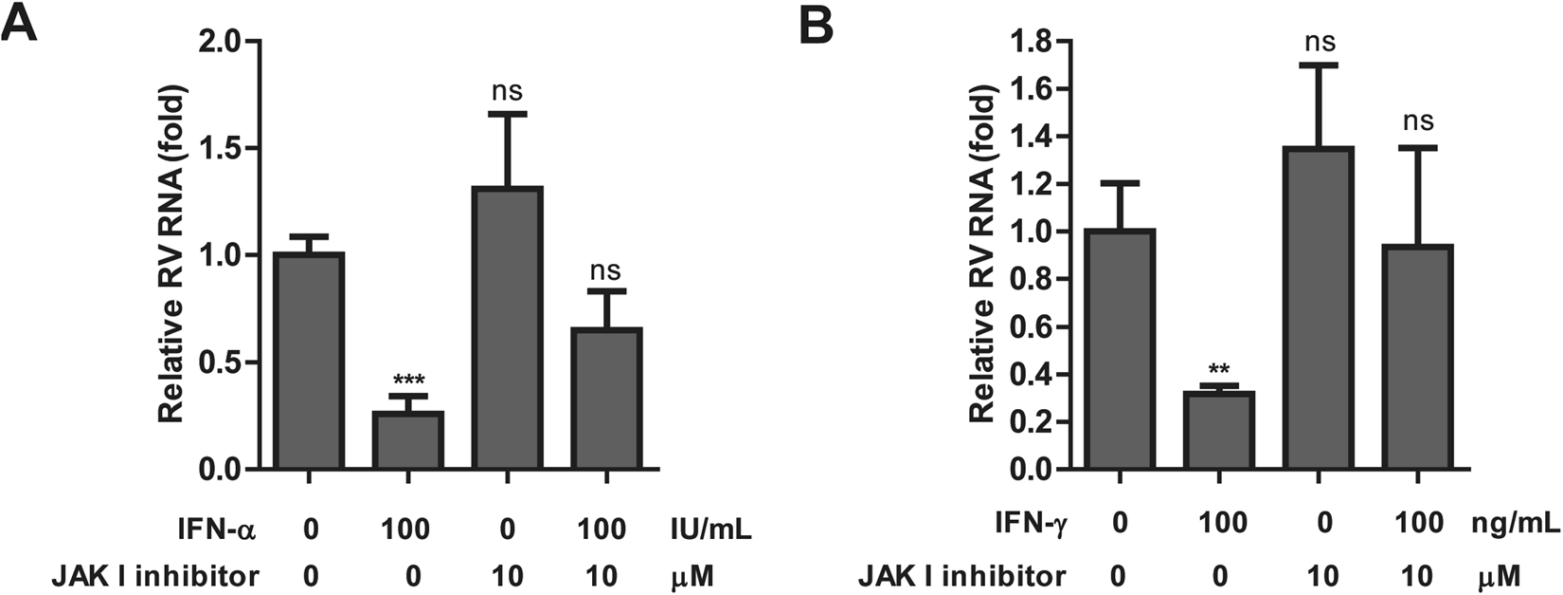
JAK I inhbitor block the anti-RV effects of IFNα and IFNγ. Caco2 cells were first infected with RV SA11 for 60 minutes. After four times washing, IFNα or IFNγ and *Pan-*JAK I inhibitor were added simultaneously to SA11-infected Caco2 cells and then cultured for 48 hours. *Pan-*JAK I inhibitor (10 μM) can block the anti-RV effects of IFNα 100 IU/mL (**A**) and IFNγ 100 ng/mL (**B**) in Caco2 cells. (n = 3 independent experiments with 2–4 replicates each) Data were presented as means ± SEM., *P < 0.05; **P < 0.01; ns, not significant.

The ISG induction by type I and III IFNs is mediated via a similar pathway involving ISGF3 complex. For type II IFN, its signaling pathway involves phosphorylation and dimerization of STAT1 to form IFNγ activation factor (GAF)^9^. To investigate the role of JAK-STAT signaling pathway in the anti-RV effects of IFNs, we used JAK I inhibitor that predominantly inhibit JAK1 protein, the upstream element that is responsible for STAT1 and STAT2 phosphorylation. As expected, JAK I inhibitor (10 μM) efficiently blocked IFNα- and IFNγ-induced ISG expression in Caco2 cells (Supplementary Figs S7 and S8). Consistently, JAK I inhibitor treatment abolished the anti-RV effects of IFN-α and IFNγ in Caco2 cell lines (Fig. 7A,B, repectively). These data clearly indicate an important role of JAK-STAT pathway in mediating the anti-RV effects of IFNs.

## Discussion

The *in vitro* study of RV biology was mainly based on the conventional two-dimensional (2D) cell culture system of intestinal carcinoma-derived cell lines, including Caco2 and HT29 cell lines^14,29–31^. They are homogenous immortalized cell lines that functionally mimic the real biological processes in humans. However, they are lacking three-dimensional (3D) details of the human intestine *in vivo*^32^. Recently, 3D models of primary intestinal organoids were developed for studying RV biology which better recapitulate the architecture and cellular composition of the human intestine^33–35^. Intestinal organoids contain heterogenous and non-transformed cell types, including enterocytes, enteroendocrine cells, goblet cells, Paneth cells and stem cells^35^. Therefore, they enable us to investigate individual-specific response associated with histo-blood group antigen (HBGAs) profiles and microbiome diversity^35^.

Transcriptional analysis of SA11-infected Caco2 cells revealed that RV predominantly induced type III (IFNλ1) rather than type I (IFNα and IFNβ) IFN responses. In other intestinal epithelial cells, such as HT29, RV infection induces type I IFN response (IFNβ) and subsequently regulates ISG expression^29,30,36^. However, our findings were consistent with previous studies in human intestinal enteroids (organoids) infected with human RV strains in which predominant type III IFN responses (IFNλ1 and IFNλ2) were observed^34^. The relatively low induction of type I IFNs in these human epithelial cells was also consistent with studies in murine RV^37^. Following murine RV infection, type I IFN response was mainly produced by immune (hematopoietic) cells, not epithelial cells^37^. A predominant type III IFN response was also observed in hepatocyte upon HCV^38^ and HEV infection^39^. However, both type I and III IFN were similarly induced following influenza virus infection in lung epithelial cells^40^. Thus, all these findings suggest that preferential induction of type I and/or type III IFN response is virus- and cell type-specific and reflects the complex regulation of type I and III IFN induction following viral infections.

Despite a clear induction of type III IFN responses, our IFN production bioassay found undetectable levels of IFN proteins in the (conditioned) culture medium from SA11-infected Caco2 cells (Fig. 2A,B). Consistently, we did not observe ISG upregulation in SA11-infected Caco2 cells (Fig. 2C), indicating an absence of secreted IFN to stimulate ISG expression in an autocrine and/or paracrine manner. Further analysis by inhibiting downstream IFN signaling using JAK inhibitors demonstrated that RV replication levels were not altered. In human intestinal enteroids (organoids) infected with human RV strains, type III IFN induction was followed by stimulation of type III-dependent ISGs^34^. These discrepancies may be due to different RV strains as well as different cellular compositions of both models. However, despite this ISG induction, blockade of type I and III IFN receptor had no effects on RV replication^34^. Altogether, these findings indicate that endogenously produced IFNs (if any) were not sufficient to limit RV replication, eventhough they were able to induce ISGs in intestinal organoid models. These findings also suggest the ability of RV to subvert innate immune responses. It is known that RV have multiple ways to blunt innate IFN responses^12^. It has been shown that RV nonstructural protein 1 (NSP1) interacts with IRF3 to promote its degradation, leading to attenuation of IFN induction^41^.

While previous studies mainly focused on the role of STAT1 in RV replication^15,42^, here we highlighted the role of basal IFN signaling in constraining RV infection. Individual knockdown of ISGF3 component, i.e. STAT1, STAT2 and IRF9, led to an elevated level of RV replication. Similarly, STAT1 knockout (KO) mice shed a significantly higher titer of RV than wild-type (WT) controls^42^. STAT1 was also shown to protect against lethal challenge of murine norovirus infection in mice^43^. In dengue virus (DENV)-infected mice, STAT2 was essentially required to protect against DENV-mediated diseases independently of STAT1^44^. Our findings were also consistent with our previous *in vitro* studies demonstrating that unphosphorylated ISGF3 complex was responsible to maintain basal transcription of ISGs in the absence of IFN stimulation to provide a “combat-ready” antiviral state in the susceptible host^27^. Thus, basal IFN signaling as maintained by its essential component, including STAT1, STAT2 and IRF9, are pivotal to restrict RV replication.

While endogenous IFN response was not able to reduce RV replication, exogenous IFN treatment was effective to limit RV replication. We demonstrated that all three types of IFNs have notable antiviral effects against simian RV SA11 both in Caco2 cells and human organoid. Patient-specific organoid lines have promising implications in personalized medicine. Noteworthy, our study employed organoids derived from only two individuals. In this aspect, using organoids derived from several numbers of patients would be much better in recapitulating inter-individual variations, including HBGAs profiles, microbime diversity and genetic background^32,35^.

We have also successfully cultivated four human-derived RV strains from acute diarrhea patients. Treatment with representative type I (IFNα), II (IFNγ) and III (IFNλ1 and IFNλ3) IFNs showed various sensitivity of human RV to IFNs, in which more pronounced inhibition was observed with type I and II rather than type III IFN treatment. Previous studies using human organoid models, type I IFN was more effective than type III IFN to suppress human RV replication^34^. However, conflicting results were found from *in vivo* studies about the relative contributions of type I and III IFNs during RV infections^25,26,45^. Type I IFN response plays a functional role to limit extra-intestinal spread in the mesenteric lymph node (MLN)^45^. On the other hand, RV has the capacity to attenuate the antiviral actions of IFNs^14^. Our study therefore suggests that human RV may differentially adapt in homologous host.

Since many previous studies mainly focused on type I and III IFNs, our study highlights the role of type II IFN (IFNγ) in limiting RV replication. Previous studies showed that IFNγ level in the serum was significantly higher in children with RV diarrhea than those of control children^17^. In our study, transcriptional analysis showed that IFNγ mRNA level was not detectable from SA11-infected Caco2 cells (data not shown). However, it has been shown that IFNγ was produced from human peripheral blood mononuclear cells (PBMCs) stimulated with RV^46^. Consistently, the level of IFNγ gene expression as well as secreted level in the supernatant of PBMCs were significantly elevated in children with RV diarrhea as compared with controls^18,47^. These findings suggest that immune cells, and not epithelial Caco2 cells, were responsible for IFNγ production upon RV infection.

RV-specific CD4+ and CD8+ IFNγ+ T cells were detected in the peripheral blood of RV-infected children and adults^48,49^. IFNγ producing T cells were also observed following experimental vaccines in animals and associated with disease protection^21,50,51^. It was previously shown that IFNγ inhibit RV entry into Caco2 cells^20^. In our study, we found that human IFNγ significantly reduced RV replication, suggesting that IFNγ can inhibit RV infection at various steps of the life cycle in the infected cells. It was suggested that IFNγ responses critically determine the severity of RV diseases in children^52^.

ISGs are the ultimate effectors of IFN-mediated antiviral responses. Based on our findings, all three types of IFNs effectively induced a panel of well-known anti-viral ISGs both in Caco2 cells and in human organoids. However, further studies are needed to identify specific anti-RV ISGs to improve our understanding of immunity against RV infections. In conclusions, our study describes the role of both endogenous and exogenous IFN in RV infection, as well as the role of both basal and activated IFN signaling in limiting RV infection. These knowledge shall contribute to the better understanding of RV-host interactions and therapeutic development.

## Material and Methods

### Reagents

Type I human recombinant IFN alpha 2a (IFNα; Thermo Scientific) and IFN beta 1a (IFNβ; Sigma-Aldrich; Catalog Number 14151); Type II IFN gamma (IFNγ; BioLegend; Catalog# 570202), and Type III IFNs IL29 (IFNλ1; Abnova), IL28A (IFNλ2; Abnova) and IL28B (IFNλ3; Abnova) were dissolved in culture medium. Stocks of Jak inhibitor I (Santa Cruz Biotech, CA) was dissolved in DMSO (Sigma-Aldrich, St Louis, MO, USA) with a final concentration of 5 mg/mL. Anti-STAT1 antibody (#9172) was purchased from Cell Signaling Technology. IRF9 antibody was obtained from LSBio (Life Span BioSciences, Inc). β-actin and STAT2 (sc-476) antibodies were purchased from Santa Cruz Biotechnology. Anti-rabbit or anti-mouse IRDye-conjugated antibodies were used as secondary antibodies for western blotting (Stressgen, Victoria, BC, Canada).

### Viruses

Simian RV SA11, a broadly used laboratory strains, was employed. SA11 RV used in this study was prepared as described previously^53^. RV genome copy numbers were determined by quantitative real-time polymerase chain reaction (qRT-PCR). A plasmid template was used to generate a standard curve by plotting the log copy number versus the cycle threshold (*C*_*T*_) as previously described^33^.

Human-derived RV strains (G1P[8]) were obtained from fecal samples of four RV patients and stored at −80 °C freezer (the Erasmus MC Biobank, Department of Viroscience, Erasmus Medical Center, Rotterdam). These samples were collected during diarrhea period and tested negative for enterovirus, parechovirus, norovirus genogroup I and II, adenovirus, astrovirus and sapovirus by qRT-PCR. The patient characteristics were shown in Supplementary Table S1.

### Cell and human primary intestinal organoid culture

Caco2 cell line (human caucasian colon adenocarcinoma ECACC) was cultured in Dulbecco’s modified Eagles’s medium (DMEM; Lonza, Verviers, Belgium) containing 20% (vol/vol) heat-inactivated fetal calf serum (FCS; Sigma-Aldrich, St. Louis USA), 100 U/mL penicillin and streptomycin (Gibco, Grand Island, USA). The cells were maintained in 5% CO_2_ at 37 °C in a humidified incubator.

Organoid culture was performed as described previously^33^. Briefly, intestinal tissues taken from biopsy were vigorously shaken in 8 mM EDTA for 15 min at 4 °C. The EDTA solution was then discarded. Loosened crypts were collected by pipetting the solution up and down for 8–10 times through a 10 mL pipette and transferred into a 50 mL tube (Greiner Bioone, the Netherlands). The biopsies were repeatedly used for crypts collection (2–3 times). Crypt suspensions were pooled and centrifuged at 300 g for 5 min. The crypt pellets were resuspended in 2 mL complete medium containing growth factors CMGF-: advanced DMEM/F12 supplemented with 1% (vol/vol) GlutaMAX™ Supplement (Gibco, Grand island, USA), 10 mM HEPES. The crypts were collected by centrifugation at 130 g for 5 min at 4 °C, suspended in matrigel (Corning, Bedford, USA) and placed in the center of a 24-well plate (40 μL per well). After the matrigel had solidified (15 min at 37 °C), organoids were maintained in culture medium at 37 °C, 5% CO_2_. Culture medium was refreshed every 2–3 days, and organoids were passaged every 6–7 days.

### Inoculation of SA11 and human-derived RV strains and treatment

Caco2 cells cultured in T75 flask were suspended and subsequently seeded into 48-well plate (5 * 10^4^ cells/well) in DMEM complemented with 20% (vol/vol) FCS and 100 IU/mL penicillin-streptomycin. After 2–3 days of culture, culture medium was removed when the cell confluence was about 80%. The cell layers were then washed twice with 500 μL PBS. Serum-free DMEM medium (100 μL) containing 5 μg/mL of trypsin (Gibco, Paisley, UK) and SA11 RV were added and incubated for 60 min at 37 °C with 5% CO_2_ to allow efficient infection, followed by four times washing with PBS (500 μL each) to remove free virus particles. Subsequently, culture medium containing 5 μg/mL of trypsin (and indicated treatments) were added to the infected cells and incubated for 24 or 48 hours at 37 °C with 5% CO_2_.

For organoid infection, SA11 RV (contain 5000 genome copies) was first activated with 5 μg/mL of trypsin at 37 °C with 5% CO_2_ for 30 minutes. Subsequently, organoids were infected with the activated SA11 for 60 minutes at 37 °C with 5% CO_2_, followed by four times washing with PBS to discard the free viruses. Organoids were then aliquoted into 48-well plates that have been coated with 20% (vol/vol) Collagen R Solution (SERVA, Heidelberg, Germany) and maintained in culture medium containing indicated treatments at 37 °C with 5% CO_2_.

### Quantitative real-time polymerase chain reaction (qRT-PCR)

Total, intracellular or extracellular (secreted) RNA was isolated by using the Machery-NucleoSpin RNA II kit (Bioke, Leiden, The Netherlands) and quantified by a Nanodrop ND-1000 spectrophotometer (Wilmington, DE, USA). cDNA was made from total RNA using a cDNA Synthesis Kit (Takara Bio Inc, Shiga, Japan) with random hexamer primers. qRT-PCR of RV RNA and genes of interest were performed with a SYBRGreen-based real-time PCR (MJ Research Opticon, Hercules, CA, USA) according to the manufacturer’s instructions with the StepOnePlus System (Thermo Fisher Scientific Life Sciences). Glyceraldehyde-3-phosphate dehydrogenase (GAPDH) gene was used as a housekeeping gene to normalize (relative) gene expression using the 2^−ΔΔCT^ formula. All primers used in this study are listed in Supplementary Table S2.

### Lentiviral vector production and transfection assays

Lentiviral pLKO knockdown vectors (Sigma–Aldrich) targeting STAT1, STAT2 and IRF9 or scrambled control, were obtained from the Erasmus Center of Biomics. All shRNA sequences are listed in Supplementary Table S3. The lentiviral vectors were produced in human embryonic kidney epithelial cell line HEK 293 T cells as previously described^54^. The shRNA vectors exerting optimal gene knockdown were selected. To generate the gene knockdown cells, Caco2 cells were transduced with the lentiviral vectors and subsequently selected by adding puromycin (8 μg/mL; Sigma) to the culture medium. Knockdown and control Caco2 cells were infected with RV as previously described.

### IFN production bioassay

The IFN production bioassay was performed to detect secreted IFN proteins in the culture medium as described previously^55^. Briefly, the culture (conditioned) medium derived from control and SA11-infected Caco2 cells (48 hours) were collected and filtered through 0.45 μm pore size membrane. Two luciferase reporter models which are extremely sensitive to IFN treatments were employed. Huh7.5-ET-Luc luciferase model is a hepatitis C virus (HCV) replicon (I389/NS3–3V/LucUbiNeo-ET) in which the HCV-related firefly luciferase activity (HCV-luc) can be potently inhibited by a low concentration of IFN-α treatments. Huh7-ISRE-luc is a luciferase reporter model in which the firefly luciferase gene was driven by multiple IFN-stimulated response elements (ISRE) promoter. In this model, the firefly luciferase activity can be potently stimulated by a low concentration of IFN-α treatment. Therefore, these two luciferase models can be employed to sensitively detect the presence of IFN proteins in the conditioned medium. Huh7 HCV-luc and ISRE-luc cells were cultured in DMEM supplemented with 10% FCS (vol/vol), 100 U/mL penicillin and streptomycin. For Huh7 HCV-luc, 250 μg/mL G418 (Sigma-Aldrich) was added to the culture medium.

### Western blot assay

Cultured cells were lysed in Laemmli sample buffer containing 0.1 M dithiothreitol (DTT) and heated for 5 min at 95 °C. Cell lysates were subjected to 10% sodium dodecyl sulfate polyacrylamide gel electrophoresis (SDS-PAGE) for 100 min running at 110 V. The proteins were transferred onto polyvinylidene difluoride (PVDF) membrane (Immobilon-FL) for 1.5 hours with an electric current of 250 mA. Subsequently, the membrane was blocked with a mixture of 2.5 ml of blocking buffer (Odyssey) and 2.5 ml of PBS containing 0.05% Tween 20 for 1 hour at room temperature. This was followed by an overnight incubation with the indicated primary antibody (1:1000 dilution) at 4 °C. The membrane was then washed three times, followed by incubation for 1 hour with IRDye-conjugated secondary antibody (1:5000 dilution) at room temperature. After washing three times, the protein bands were detected with the Odyssey 3.0 Infrared Imaging System. The intensity of the immunoreactive bands of blotted proteins was quantified by the Odyssey V3.0 software.

### Immunofluorescence microscope assay

Caco2 cells were seeded on glass coverslips. After SA11 infection for 48 hours, cells were washed with PBS, fixed in 4% PBS-buffered formalin for 10 mins and blocked with tween-milk-glycine medium (PBS, 0.05% tween, 5 g/L skim milk and 1.5 g/L glycine). Samples were incubated with anti-rotavirus (ab181695) antibody (Abcam) overnight at 4 °C. Subsequently, samples were incubated with 1:1000 dilutions of Alexa Fluor^TM^ 594 goat anti-mouse secondary antibodies (Invitrogen). Nuclei were stained with DAPI (4,6-diamidino-2-phenylindole; Invitrogen). Images were detected using immunofluorescence microscope.

### MTT assay

10 mM 3-(4,5-dimethylthiazol-2-yl)-2,5-diphenyltetrazolium bromide (MTT) (Sigma) was added to Caco2 cells seeded in 96-well plates at indicated time points. The cells were incubated at 37 °C with 5% CO_2_ for 3 hours. The culture medium was then removed and 100 μl of dimethyl sulfoxide (DMSO) was added to each well. The absorbance of each well was read on the microplate absorbance readers (BIO-RAD) at wavelength of 490 nm.

### Study Approval

Human intestinal tissue were obtained during surgical resection. A written informed consent was signed by the volunteers or patients who agreed to participate. The study was approved by the Medical Ethical Committee of the Erasmus Medical Center (Medisch Ethische Toetsings Commissie Erasmus MC), and all experiments were performed in accordance with relevant guidelines and regulations.

### Statistical Analysis

Statistical analysis was performed using the nonpaired, nonparametric test (Mann-Whitney test; GraphPad Prism software, GraphPad Software Inc., La Jolla, CA). *P* values < 0.05 were considered statistically significant.

## Electronic supplementary material

Supplementary Information
