# Supplementary material for: Basal interferon signaling and therapeutic use of interferons in controlling rotavirus infection in human intestinal cells and organoids
Source: Sci Rep. 2018 May 29;8:8341. doi: 10.1038/s41598-018-26784-9 (PMC5974418; doi:10.1038/s41598-018-26784-9)
Supplement: Supplementary file 1 — Supplementary Information [file 41598_2018_26784_MOESM1_ESM.doc]

**Basal interferon signaling and therapeutic use of interferons in controlling rotavirus infection in human intestinal cells and organoids**

**Mohamad S. Hakim, Sunrui Chen, Shihao Ding, Yuebang Yin, Aqsa Ikram, Xiaoxia Ma, Wenshi Wang, Maikel P. Peppelenbosch, Qiuwei Pan**

**A**

**B**

**Fig. S1**

**Supplementary Figure S1. Efficient replication of rotavirus SA11 in Caco2 cells as demonstrated by qRT-PCR.** Intracellular **(A)** and secreted (extracellular) levels **(B)** of rotavirus SA11 were detected and quantified by qRT-PCR at 0 (before virus inoculation) as well as at 1, 24 and 48 hours after inoculation. For intracellular RNA quantification **(A)**, RV RNA levels were shown as fold increases relative to 1 hour post infection. GAPDH was used as the housekeeping gene.


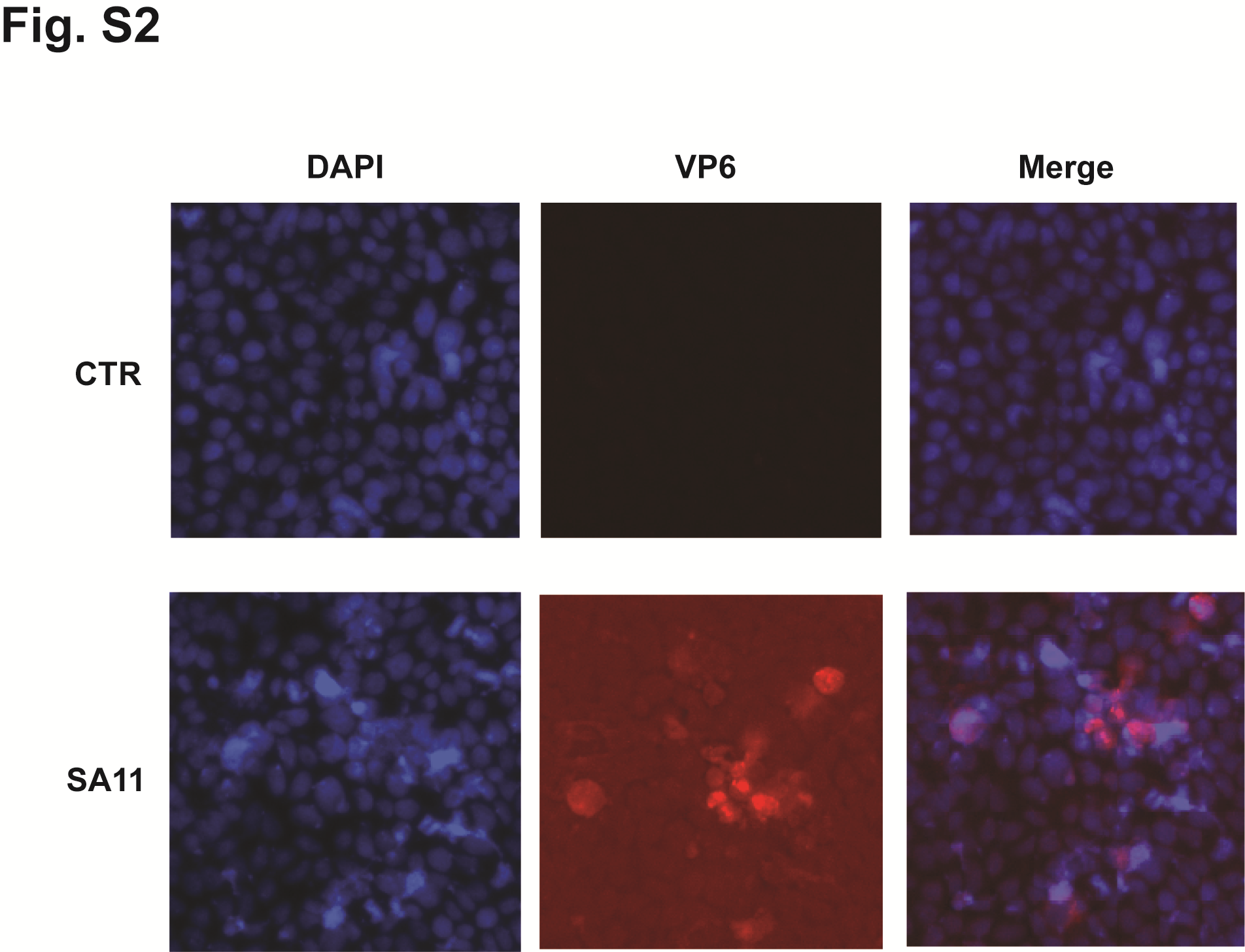


**Supplementary Figure S2. Efficient replication of rotavirus SA11 in Caco2 cells as demonstrated by immunofluorescence stainings.** Representrative immunofluorescence stainings of ab181695 (targeting VP6 rotavirus protein) (red) after 48 hours infection of Caco2 cells with rotavirus SA11. Nuclei were visualized by DAPI (blue).


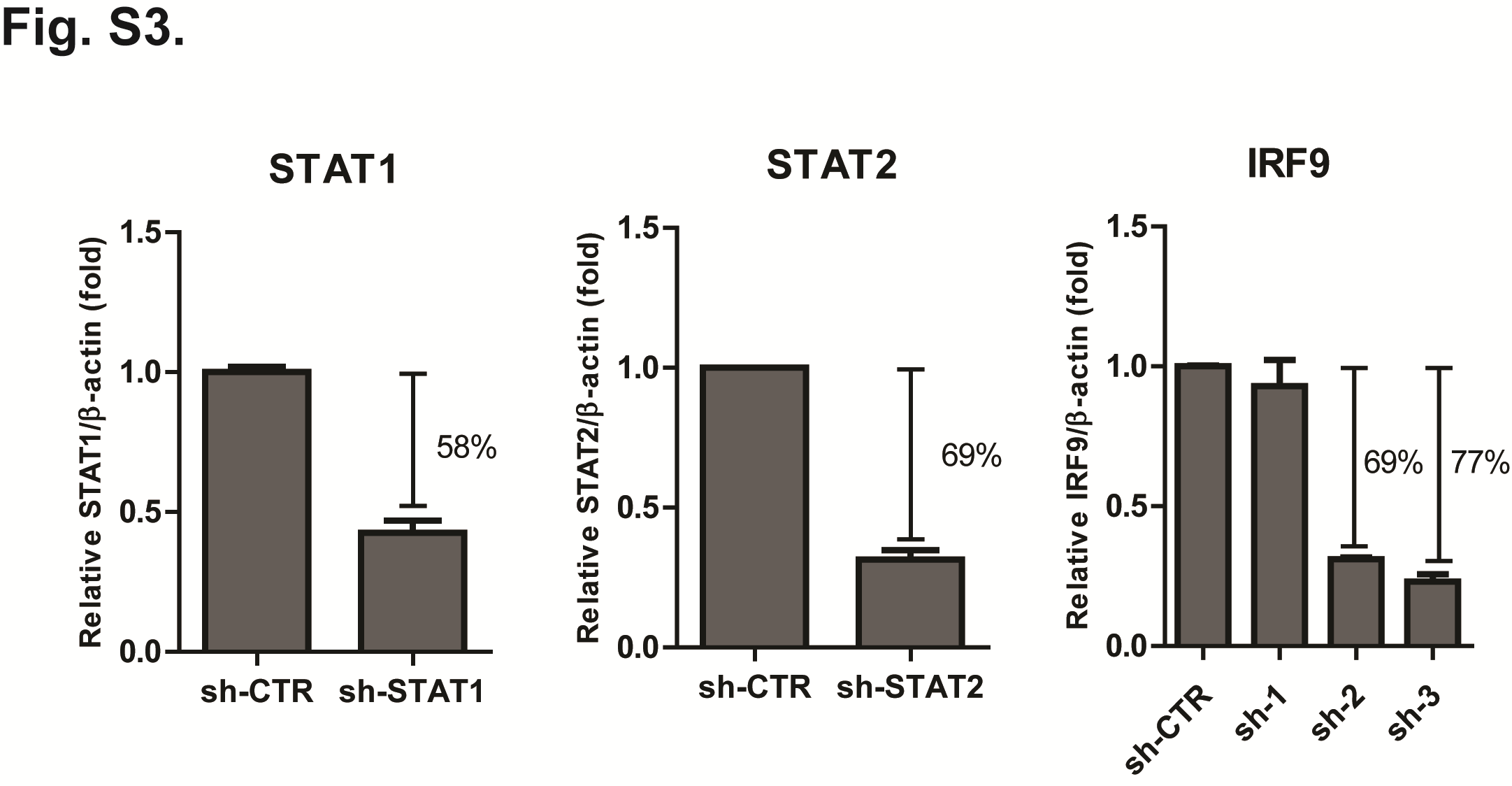


**Supplementary Figure S3.** Western blot assays showed a succesfull knockdown of STAT1 (58% reduction), STAT2 (69% reduction) and IRF9 proteins (69% and 77% reduction). β-actin served as an internal reference. (means ± SEM, n = 3).


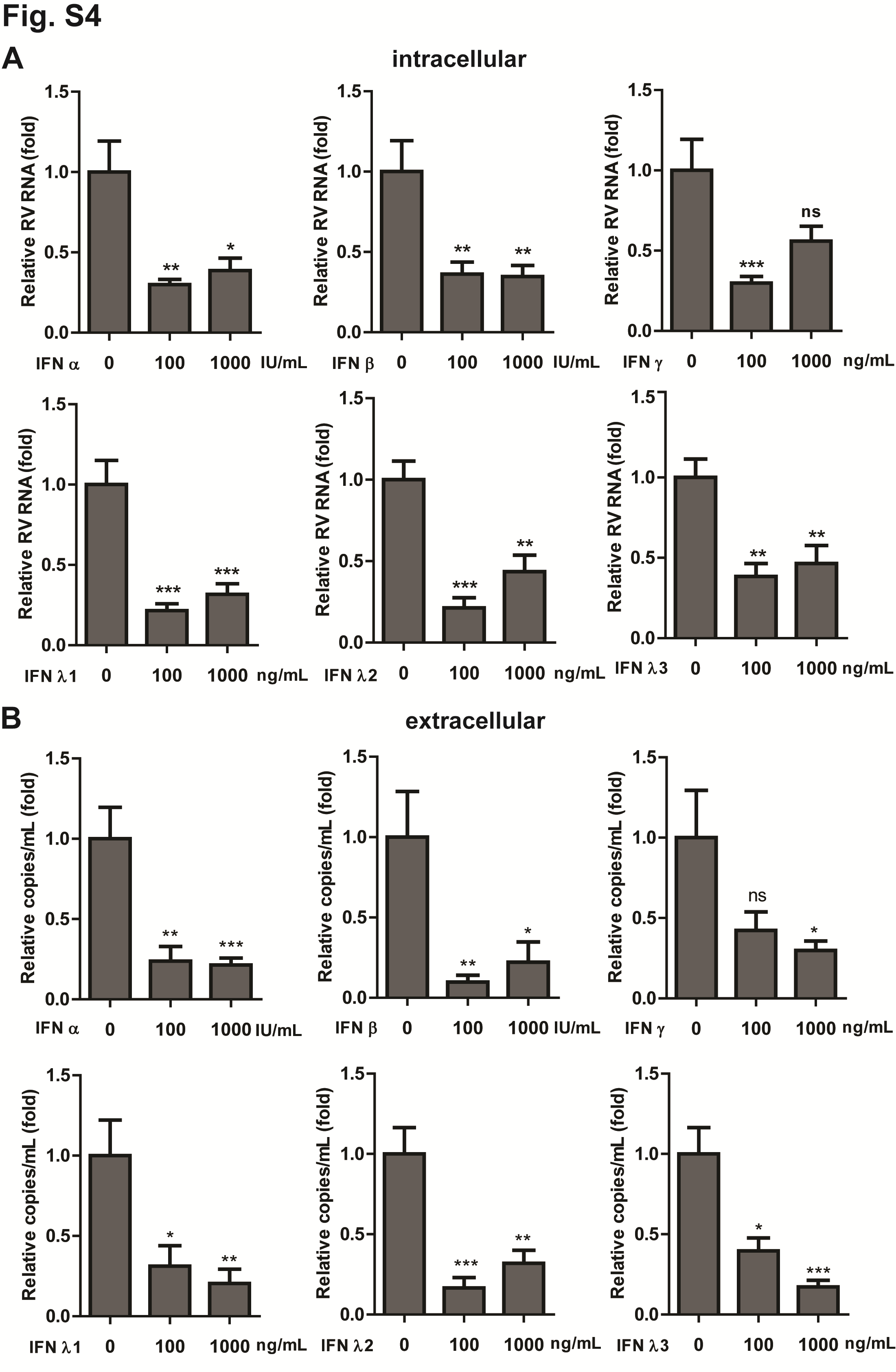


**Supplementary Figure S4. The effects of exogenous treatment of type I, II and III IFNs on RV SA11 replication in Caco2 cells.** Antiviral activities of IFNα, IFNβ, IFNγ, IFNλ1, IFNλ2 and IFNλ3 treatments against RV SA11 infection on Caco2 cells were determined by quantifying intracellular **(A)** and extracellular (secreted) **(B)** RNA levels at 48 hours post-infection. (n = 2-3 independent experiment with each of 3 replicates) Data were presented as means ± SEM., **P* < 0.05; ***P* < 0.01; ****P* < 0.001; ns, not significant.


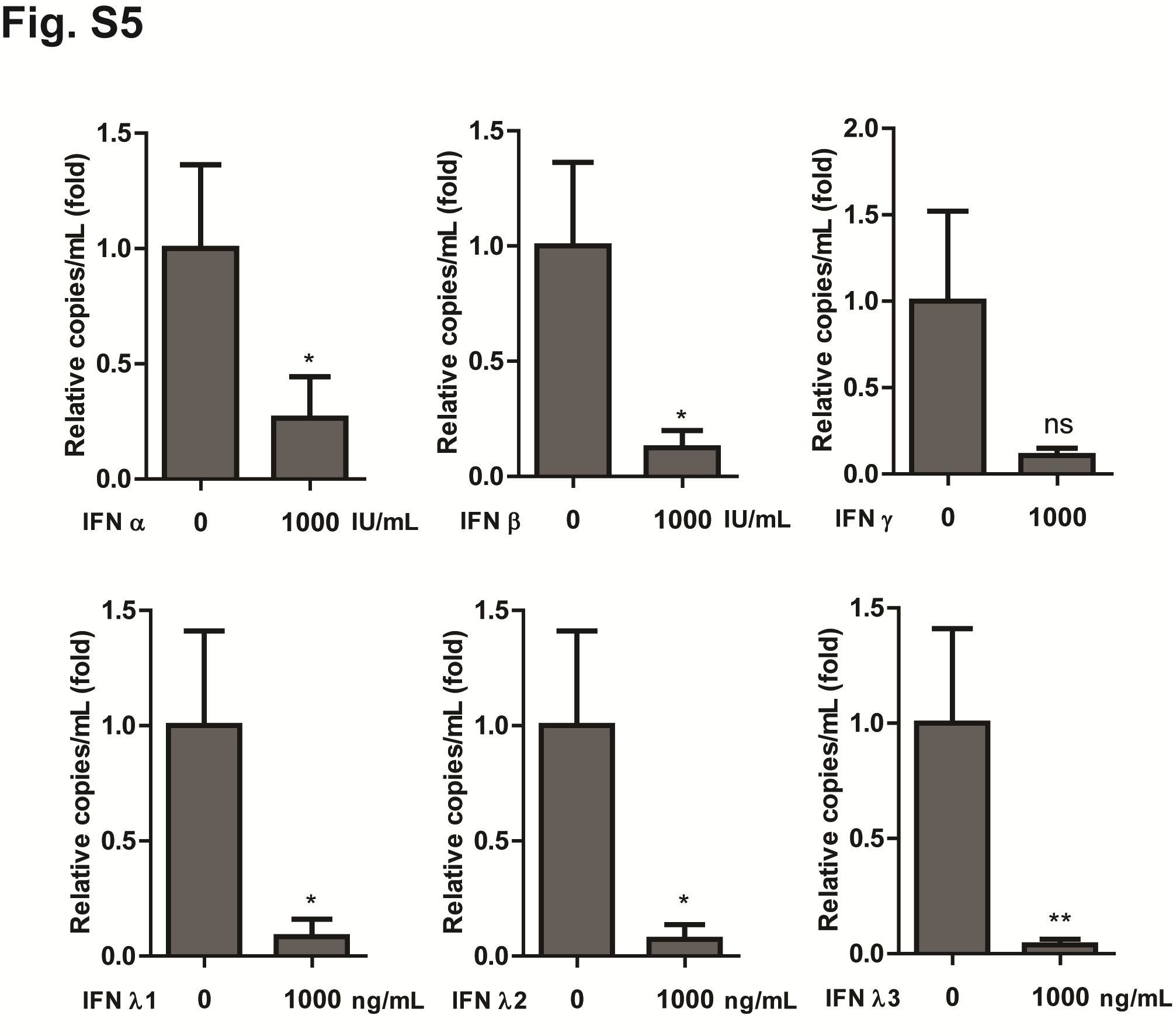


**Supplementary Figure S5. The effects of type I, II and III IFNs on extracellular level of RV SA11 in human organoids.** Antiviral effects of IFNα, IFNβ, IFNγ, IFNλ1, IFNλ2 or IFNλ3 treatment against RV SA11 infection in organoids were determined by quantifying extracellular (secreted) RNA levels at 48 hours post-infection. The organoids were derived from one individual (P1). Data were presented as means ± SEM., **P* < 0.05; ***P* < 0.01; ns, not significant. (n = 3 independent experiments with each of 2-3 replicates).


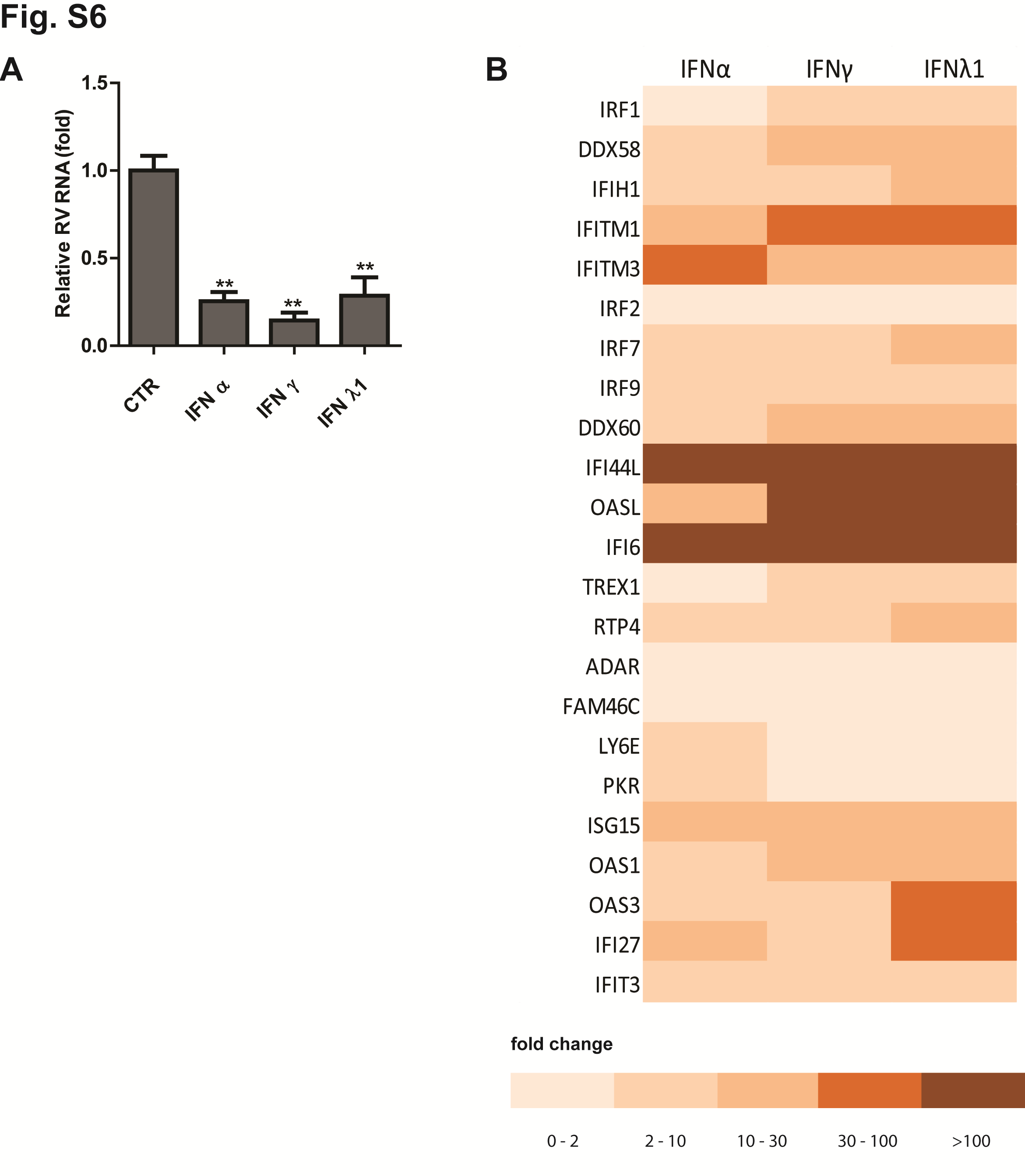


**Supplementary Figure S6. (A)** Antiviral effects of representative type I (IFNα, 1000 IU/mL), type II (IFNγ, 1000 ng/mL), and type III (IFNλ1, 1000 ng/mL) treatment against RV SA11 infection in organoids were determined by quantifying total RV RNA levels at 48 hours post-infection. The organoid was derived from individual 2 (P2). Data were presented as means ± SEM, ***P* < 0.01. (n = 3 independent experiments with each of 1-2 replicates). **(B)** Organoids (P2) were stimulated with IFNα 1000 IU/mL, IFNγ 1000 ng/mL and IFNλ1 1000 ng/mL for 24 hours. The expression levels of several ISGs were measured by qRT-PCR.


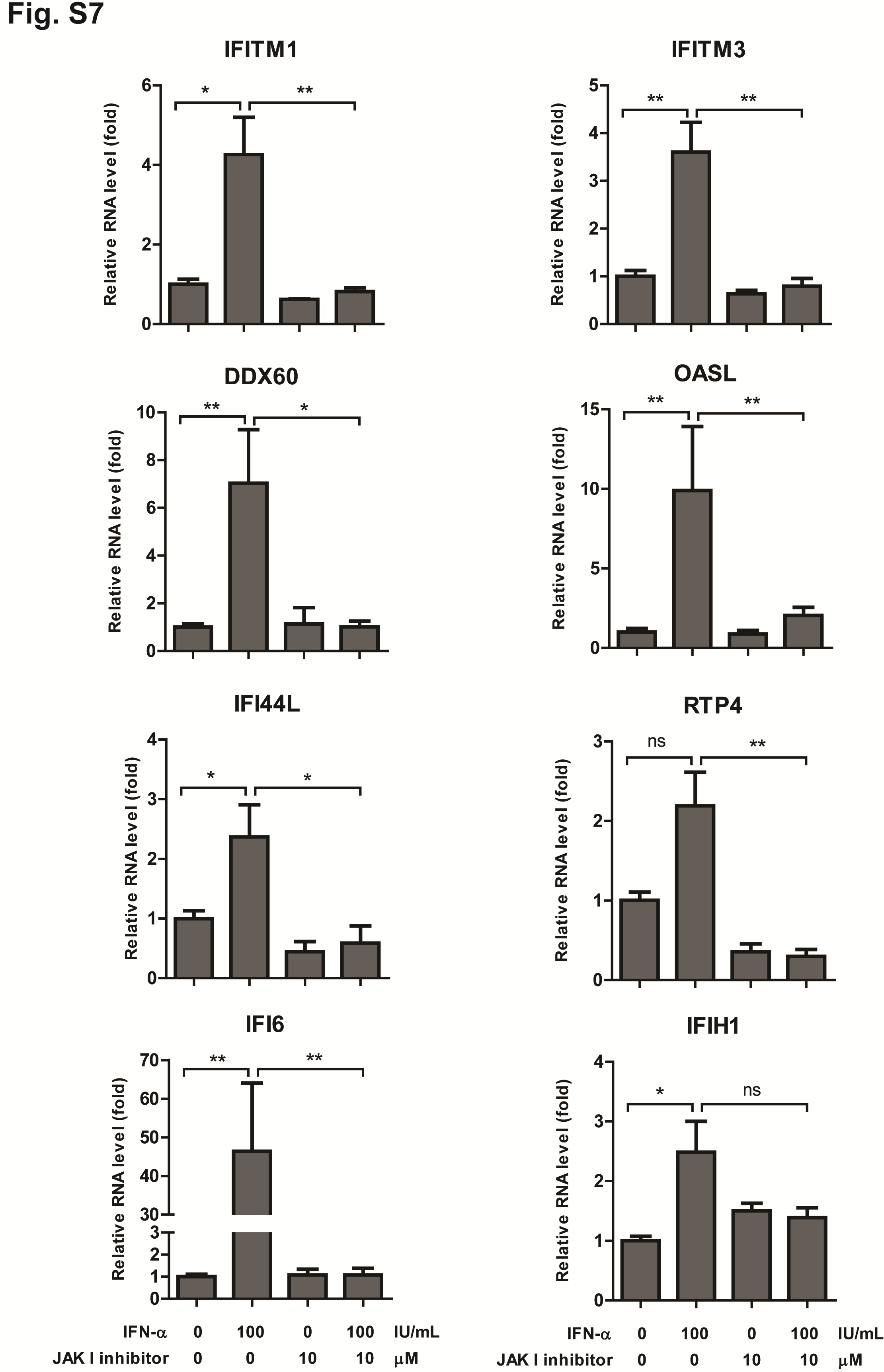


**Supplementary Figure S7. JAK I inhbitor blocks IFNα-induced ISGs in Caco2 cells.** JAK I inhibitor (10 μM) abrogated IFNα- (100 IU/mL) induced ISG expression as measured by qRT-PCR. Data were presented as means ± SEM., **P* < 0.05; ***P* < 0.01; ns, not significant. (n = 3 independent experiments with each of 2-3 replicates)


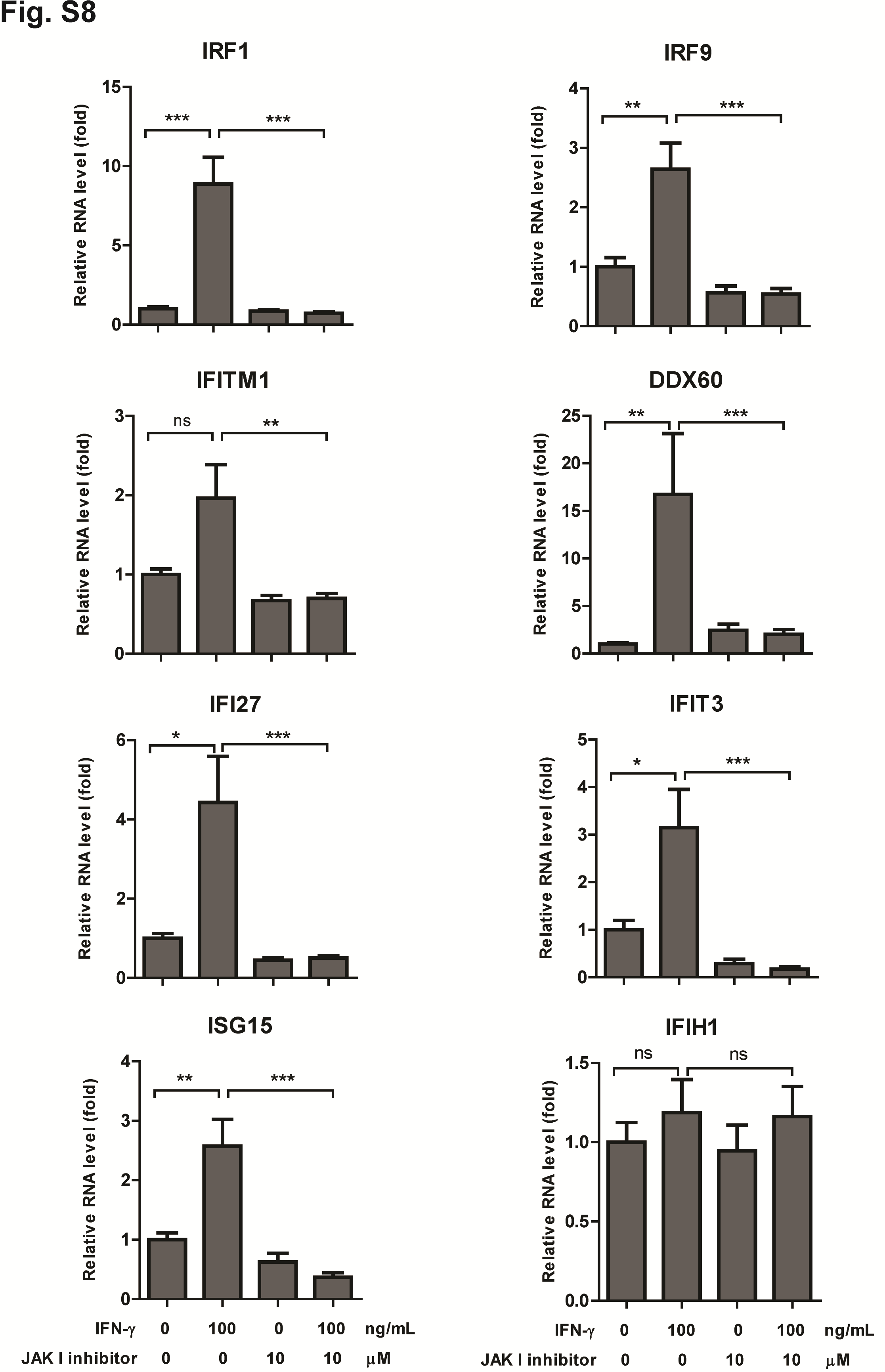


**Supplementary Figure S8. JAK I inhbitor abolishes IFNγ-induced ISGs in Caco2 cells.** JAK I inhibitor (10 μM) abrogated IFNγ- (100 ng/mL) induced ISG expression as measured by qRT-PCR. Data were presented as means ± SEM., **P* < 0.05; ***P* < 0.01; ****P* < 0.001; ns, not significant. (n = 3 independent experiments with each of 2-3 replicates)

Supplementary Table S1. Patient characteristics.

| Patient | Age (years) | Gender | Clinical symptoms | Virus Detection | | | | | | | |
| --- | --- | --- | --- | --- | --- | --- | --- | --- | --- | --- | --- |
|  |  |  |  | Enterovirus | Parechovirus | Norovirus genogroups I | Norovirus genogroups II | Adenovirus | Astrovirus | Sapovirus | Rotavirus |
| 1 | 74 | Female | Congestive heart failure, myocarditis | No | No | No | No | No | No | No | Yes |
| 2 | 27 | Female | Fever, diarrhea, nausea, vomiting | No | No | No | No | No | No | No | Yes |
| 3 | 67 | Male | Fever, stomach ache, watery diarrhea (Kidney transplant) | No | No | No | No | No | No | No | Yes |
| 4 | 1.5 | Female | Vomiting, watery diarrhea | No | No | No | No | No | No | Yes | Yes |

**Supplementary Table S2. Primers used in this study.**

| **Target Genes** | **Sequences (5’ - 3’)** |
| --- | --- |
| **Viruses** |  |
| **RV SA11-F** | TGGTTAAACGCAGGATCGGA |
| **RV SA11-R** | AACCTTTCCGCGTCTGGTAG |
| **Human RV-F** | ACCATCTACACATGACCCTC |
| **Human RV-R** | CACATAACGCCCCTATAGCC |
| **Human Genes** |  |
| **IRF1-F** | GAGGAGGTGAAAGACCAGAGCA |
| **IRF1-R** | TAGCATCTCGGCTGGACTTCGA |
| **DDX58-F** | CACCTCAGTTGCTGATGAAGGC |
| **DDX58-R** | GTCAGAAGGAAGCACTTGCTACC |
| **IFIH1(MDA5)-F** | GCTGAAGTAGGAGTCAAAGCCC |
| **IFIH1(MDA5)-R** | CCACTGTGGTAGCGATAAGCAG |
| **IFITM1-F** | GGCTTCATAGCATTCGCCTACTC |
| **IFITM1-R** | AGATGTTCAGGCACTTGGCGGT |
| **IFITM3-F** | CTGGGCTTCATAGCATTCGCCT |
| **IFITM3-R** | AGATGTTCAGGCACTTGGCGGT |
| **IRF2-F** | TAGAGGTGACCACTGAGAGCGA |
| **IRF2-R** | CTCTTCATCGCTGGGCACACTA |
| **IRF7-F** | CCACGCTATACCATCTACCTGG |
| **IRF7-R** | GCTGCTATCCAGGGAAGACACA |
| **IRF9-F** | CCACCGAAGTTCCAGGTAACAC |
| **IRF9-R** | AGTCTGCTCCAGCAAGTATCGG |
| **DDX60-F** | GGTGTTTTCACCAGGGAGTATCG |
| **DDX60-R** | CCAGTTTTGGCGATGAGGAGCA |
| **IFI44L-F** | TGCACTGAGGCAGATGCTGCG |
| **IFI44L-R** | TCATTGCGGCACACCAGTACAG |
| **OASL-F** | GTGCCTGAAACAGGACTGTTGC |
| **OASL-R** | CCTCTGCTCCACTGTCAAGTGG |
| **IFI6-F** | TGATGAGCTGGTCTGCGATCCT |
| **IFI6-R** | GTAGCCCATCAGGGCACCAATA |
| **TREX-F** | GCATCTGTCAGTGGAGACCACA |
| **TREX-R** | CAGTGGTTGTGACAGCAGATGG |
| **RTP4-F** | GACGCTGAAGTTGGATGGCAAC |
| **RTP4-R** | GTGGCACAGAATCTGCACTTGG |
| **ADAR-F** | TCCGTCTCCTGTCCAAAGAAGG |
| **ADAR-R** | TTCTTGCTGGGAGCACTCACAC |
| **FAM46C-F** | CCTTGAACAGCAGAGGAAGTTGG |
| **FAM46C-R** | GGAGATGAGGTTCAGAGTCTGC |
| **LY6E-F** | GACCAGGACAACTACTGCGTGA |
| **LY6E-R** | AAGCCACACCAACATTGACGCC |
| **PKR-F** | GAAGTGGACCTCTACGCTTTGG |
| **PKR-R** | TGATGCCATCCCGTAGGTCTGT |
| **ISG15-F** | CTCTGAGCATCCTGGTGAGGAA |
| **ISG15-R** | AAGGTCAGCCAGAACAGGTCGT |
| **OAS1-F** | AGGAAAGGTGCTTCCGAGGTAG |
| **OAS1-R** | GGACTGAGGAAGACAACCAGGT |
| **OAS3-F** | CCTGATTCTGCTGGTGAAGCAC |
| **OAS3-R** | TCCCAGGCAAAGATGGTGAGGA |
| **IFI27-F** | CGTCCTCCATAGCAGCCAAGAT |
| **IFI27-R** | ACCCAATGGAGCCCAGGATGAA |
| **IFIT3-F** | CCTGGAATGCTTACGGCAAGCT |
| **IFIT3-R** | GAGCATCTGAGAGTCTGCCCAA |
| **IFNαR1-F** | CGCCTGTGATCCAGGATTATCC |
| **IFNαR1-R** | TGGTGTGTGCTCTGGCTTTCAC |
| **IFNλR1-F** | CAGCAAGTTCTCTAAGCCCACC |
| **IFNλR1-R** | GTCATTCACGGACTCTGGTCTG |
| **IFNα-F** | GACTCCATCTTGGCTGTGA |
| **IFNα-R** | TGATTTCTGCTCTGACAACCT |
| **IFNβ1-F** | CTTGGATTCCTACAAAGAAGCAGC |
| **IFNβ1-R** | TCCTCCTTCTGGAACTGCTGCA |
| **IFN-γ-F** | GAGTGTGGAGACCATCAAGGAAG |
| **IFN-γ-R** | TGCTTTGCGTTGGACATTCAAGTC |
| **IFNλ1 (IL-29)-F** | GGAAGACAGGAGAGCTGCAACT |
| **IFNλ1 (IL-29)-R** | AACTGGGAAGGGCTGCCACATT |
| **IFNλ2/3 (IL-28)-F** | TCGCTTCTGCTGAAGGACTGCA |
| **IFNλ2/3 (IL-28)-R** | CCTCCAGAACCTTCAGCGTCAG |
| **GAPDH-F** | TGTCCCCACCCCCAATGTATC |
| **GAPDH-R** | CTCCGATGCCTGCTTCACTACCTT |

**Supplementary Table S**3. Lentiviral sh-RNA sequences used in this study.

| **Name** | **Oligo Sequences (5’ - 3’)** |
| --- | --- |
| **shSTAT1** | CCGGCGACAGTATGATGAACACAGTCTCGAGACTGTGTTCATCATACTGTCGTTTTT |
| **shSTAT2** | CCGGGCTGAGCCATAGGTCTAAATACTCGAGTATTTAGACCTATGGCTCAGCTTTTT |
| **shIRF9-1** | CCGGTTCAAGGCCTGGGCAATATTTCTCGAGAAATATTGCCCAGGCCTTGAATTTTTG |
| **shIRF9-2** | CCGGGAGACTTGGTCAGGTACTTTCCTCGAGGAAAGTACCTGACCAAGTCTCTTTTTG |
| **shIRF9-3** | CCGGCTCAGTAGTTGTCCGTGATAACTCGAGTTATCACGGACAACTACTGAGTTTTTG |

**Supplementary Figure.**

**The original picture of Fig. 3A**

**
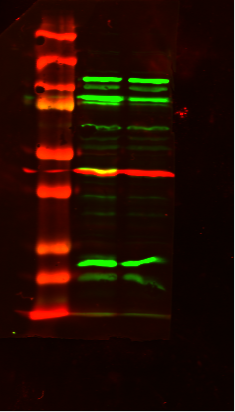
**

STAT1 91 kDa

Actin 42 kDa

**The original picture of Fig. 3B**


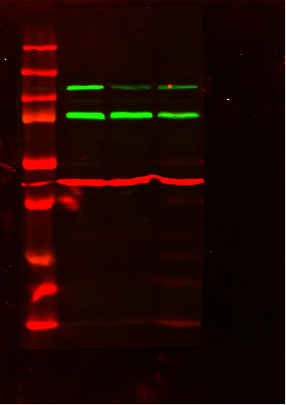


STAT2 98 kDa

Actin 42 kDa

**The original picture of Fig. 3C**

**
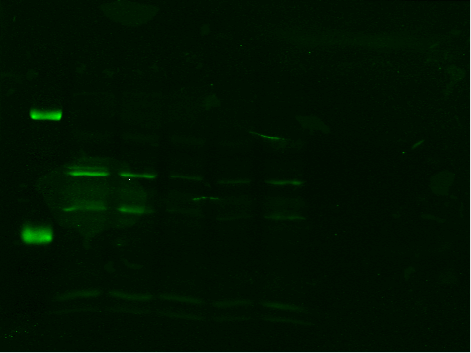
**

IRF9 44 kDa


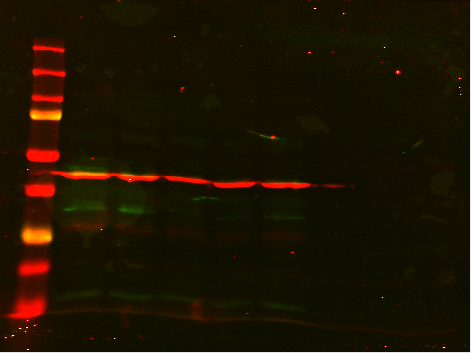


Actin 42 kDa
